# Management of Clavicle Implant Failure With Olecranon Bone Graft: A Case Report

**DOI:** 10.7759/cureus.67358

**Published:** 2024-08-21

**Authors:** Yee Sean Kong, Muhammad Shukri Muhammad Safian

**Affiliations:** 1 Orthopaedics and Traumatology, Hospital Canselor Tuanku Muhriz, Kuala Lumpur, MYS; 2 Orthopaedic Surgery, Hospital Serdang, Selangor, MYS

**Keywords:** clavicle, cortical-cancellous, autograft, autologous, bone graft, olecranon

## Abstract

Bone grafts have been important treatment tools in reconstructive orthopaedic surgery for years for traumatic osseous defects, defects resulting from resection of bone tumours, arthrodesis, and osteotomy procedures. We used an autologous olecranon bone graft to fill the bone gap due to fracture comminution in a patient with right clavicle implant failure. A 21-year-old male initially underwent primary fixation with a clavicle locking plate for a comminuted middle-third right clavicle fracture. At the follow-up visit after four weeks, the patient complained of pain over the right shoulder. Repeated radiographs revealed no evidence of union with implant failure. Revision surgery was done, and a cortical-cancellous bone graft was harvested from the ipsilateral olecranon to fill the osseous defect over the clavicle fracture site.

## Introduction

Autologous bone grafts are commonly used in orthopaedics and reconstructive surgery. Olecranon bone autograft is relatively new compared to the more widely used iliac crest, tibia, fibula, and cranial grafts. Some other graft sources in the upper limb include the proximal third of the ulna and the distal third of the radius [1]. Bone autografts can be either cancellous or cortical [2]. Cancellous autografts provide trabecular bone lined with osteoblasts for osteogenesis. However, they lack structural strength. Cortical autografts are rarely used due to donor-site morbidity, but they provide structural support and scaffold with some osteoblasts [2]. In certain cases, both cortical and cancellous can be harvested and incorporated into the recipient site. Olecranon bone grafts are easily obtained and provide versatility and superior structural properties with minimal donor-site morbidity. A study has shown excellent outcomes in treating neglected forearm fractures with olecranon bone grafts [3]. We report our clinical experience with olecranon bone grafts in a case of clavicle implant failure.

## Case presentation

**Figure 1:**
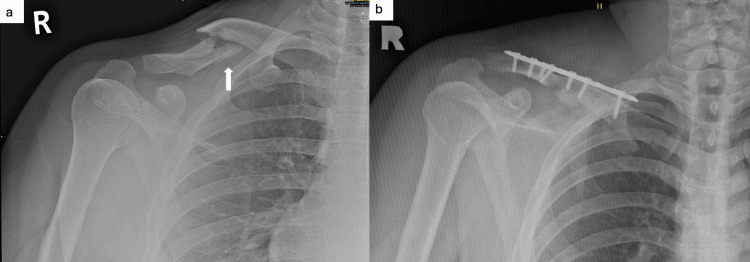
Plain radiograph of the right shoulder. (a) X-ray of the right shoulder post-trauma: comminuted fracture (white arrow) over the middle third right clavicle. (b) X-ray of the right shoulder after primary fixation: fracture fixed with a 3.5 mm straight locking plate (LCP).

We report the case of a patient with closed right clavicle implant failure who underwent revision surgery with a distal clavicle locking plate with lateral extension and olecranon bone graft. A 21-year-old male with no underlying medical illness was initially involved in a motor vehicle accident and sustained a closed comminuted right middle third clavicle fracture. Primary fixation with a 3.5 mm straight clavicle locking plate was done to treat the fracture (Figure 1).

**Figure 2:**
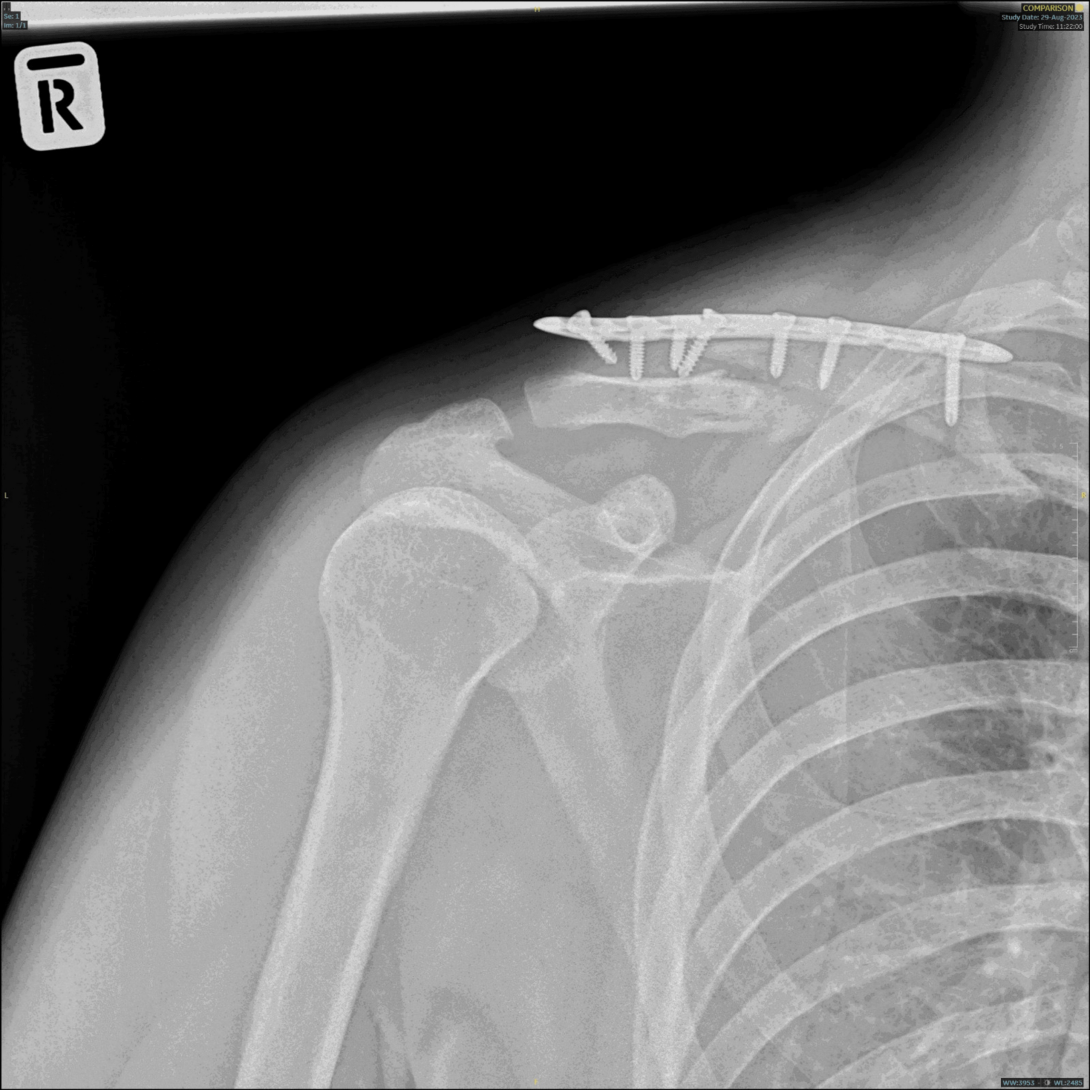
Plain radiograph four weeks after primary fixation. Screw cut out from distal clavicle with no callus formation.

Although the fracture involved the non-dominant hand, the patient heavily relied on both hands for his work as a chef. The patient was discharged well post-operatively. Upon clinic review four weeks post-op, he complained of pain at the operative side for the past few days after initiating a home-based range of motion exercises. He denied another episode of fall or injury after discharge. He sought no immediate medical treatment as he expected some post-procedural pain. Clinical examination revealed tenderness over the right clavicle region. The previous surgical wound had healed well with primary intention. There was no sign of warmth, erythema, swelling, purulent discharge, or sinus to suggest implant-associated infection. Repeated plain radiographs of the right shoulder revealed implant failure with no evidence of fracture union (Figure 2).

**Figure 3:**
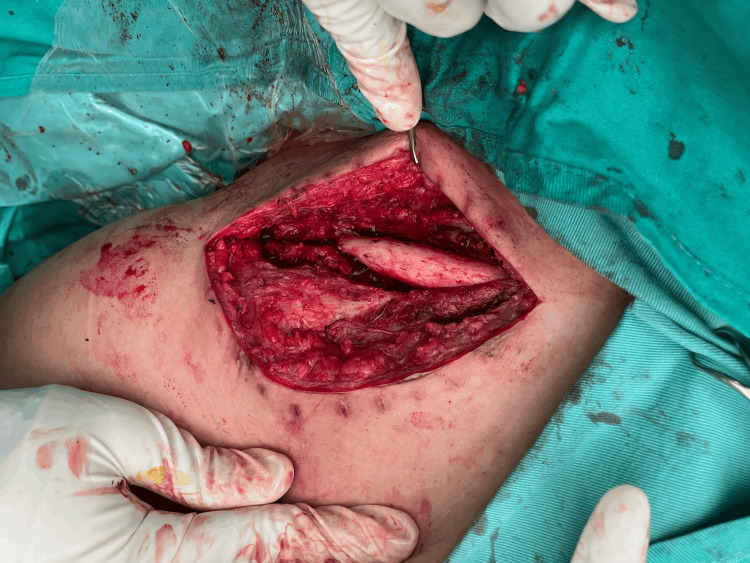
Revision surgery after skin exposure. The site revealed bone loss over the inferior aspect of the clavicle with no callus over the fracture site.

Inflammatory markers were within the normal range. The patient was counselled and informed of the need for revision surgery. One week later, the patient was subjected to revision surgery under general anaesthesia in a supine position, putting a sandbag between the medial border of the scapula and the spine. A skin incision was made over the previous surgical scar, and dissection was done layer by layer to expose the clavicle. Intraoperative examination revealed screws cut out from the lateral clavicle with the presence of fibrous tissue and 3.0 × 1.0 cm bone loss over the inferior aspect of the clavicle due to the fracture comminution (Figure 3).

**Figure 4:**
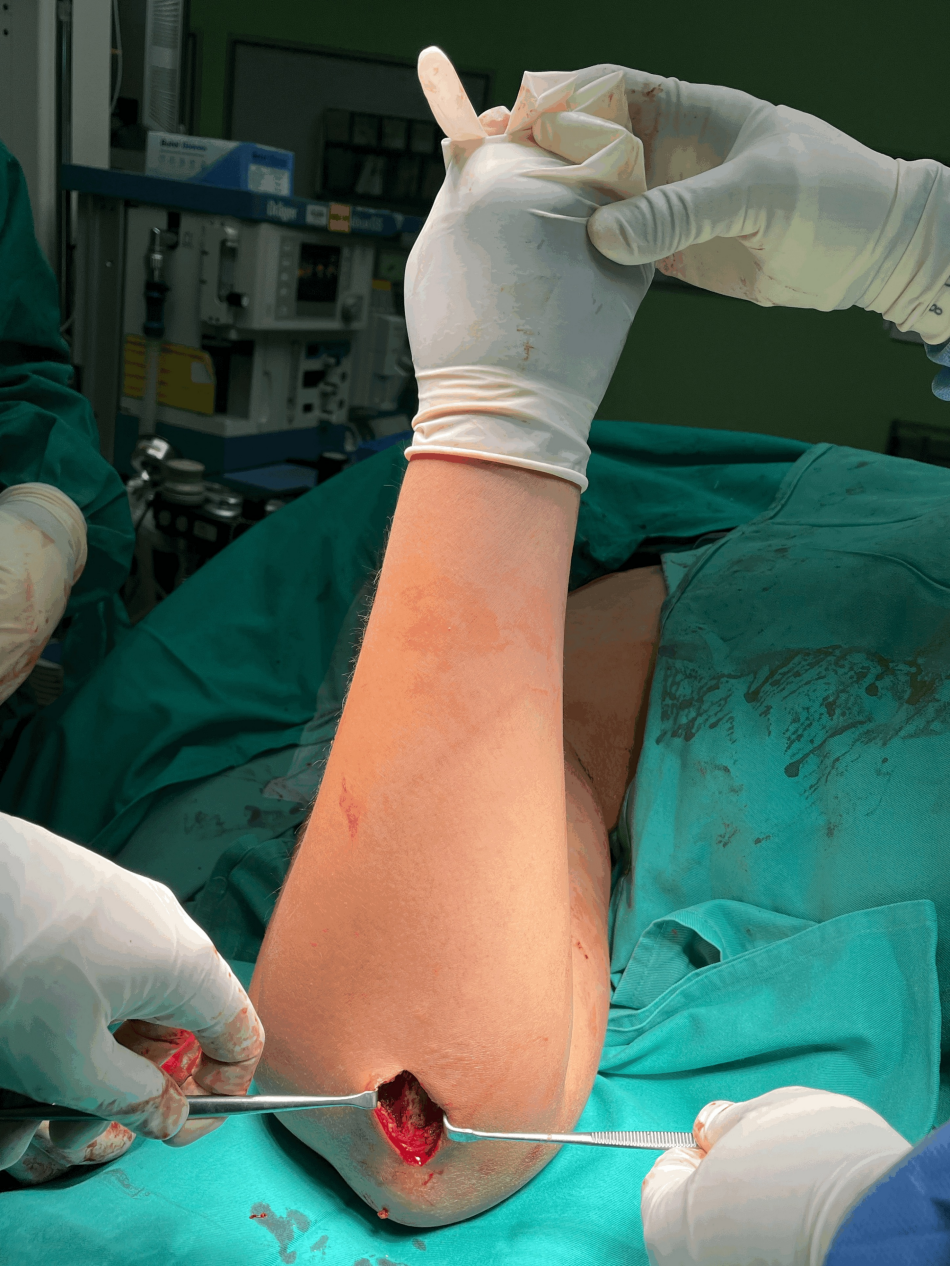
Position of the forearm during bone graft harvesting. The elbow was flexed to 90 degrees, and a 4 cm incision was made 1 cm distal to the tip of the olecranon.

Therefore, we decided to use a bone graft for bone void filling. For bone graft harvesting, a bolster was placed under the arm, and the elbow was flexed to 90 degrees for adequate exposure to the surgical field. A 4 cm incision was made 1 cm distal to the dorsal tip of the olecranon (Figure 4).

**Figure 5:**
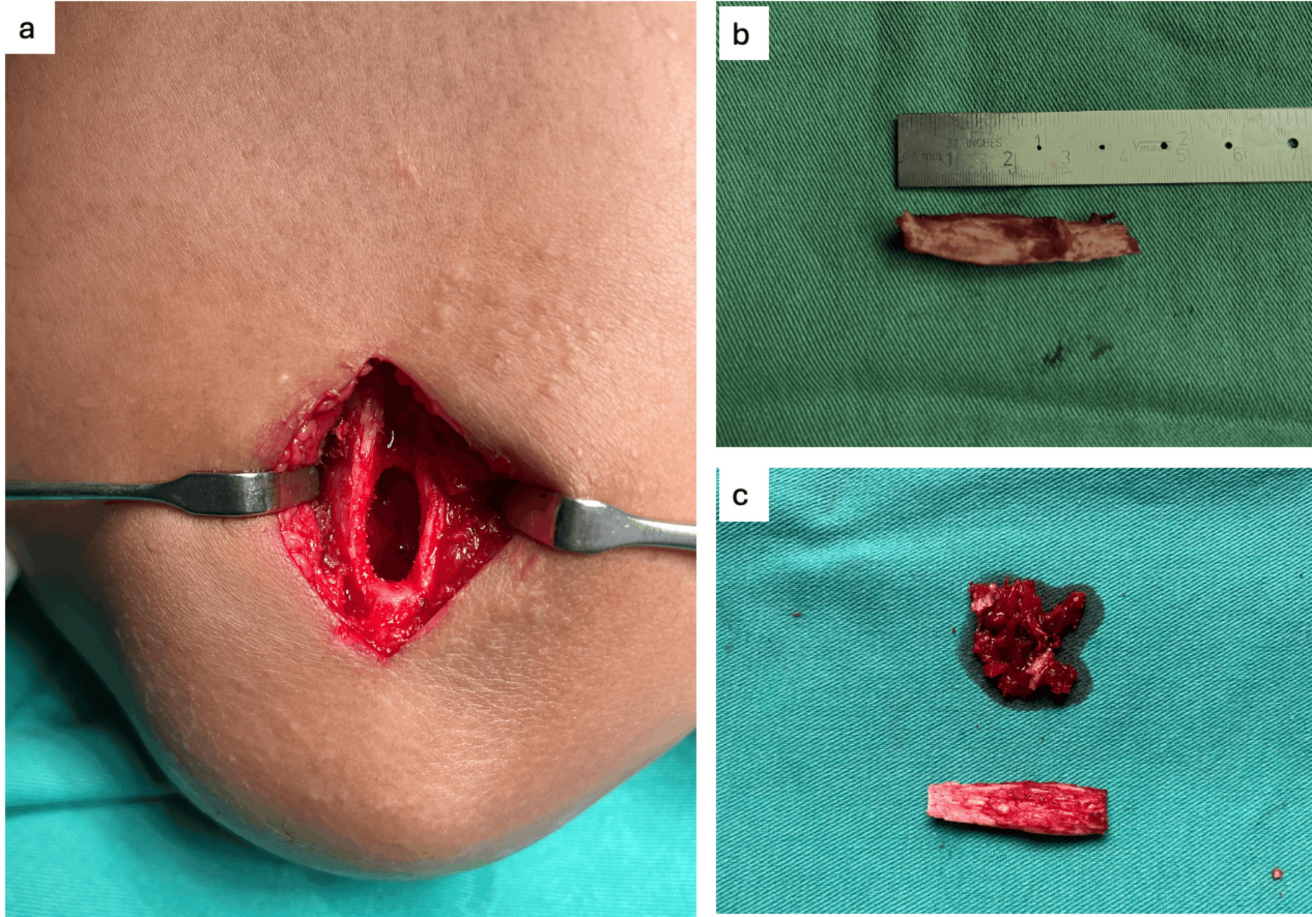
Right olecranon bone graft. (a) Cortical window made via osteotome and corticocancellous bone graft harvested. (b) A 4×1 cm of cortical bone graft obtained. (c) A 1-2 cc of cancellous bone obtained alongside cortical bone.

Subcutaneous tissue was dissected to expose the periosteum. The intended cortical window was marked over the periosteum via diathermy. A straight osteotome was used to cut through the bony cortex and create a window. About 1.0 - 2.0 cc of cancellous bone was curetted, and 4 cm × 1 cm cortical bone was harvested (Figure 5).

**Figure 6:**
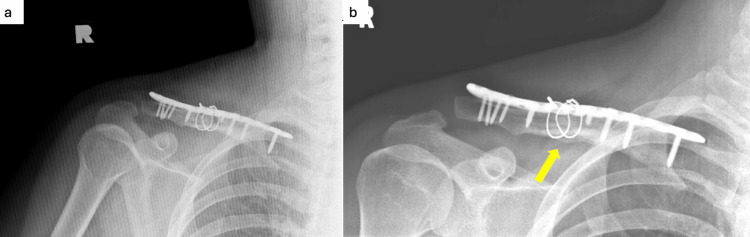
Plain radiograph of the right shoulder post revision surgery. (a) Immediate post-operative X-ray: distal clavicle locking plate inserted and cerclage wire used to hold olecranon cortical bone graft to the clavicle. (b) X-ray at four months post-op: callus formation (yellow arrow).

The olecranon donor site was packed with 10 cc bone graft chips before soft tissue closure over the proximal forearm. The clavicle fracture site was reduced and stabilised with a distal clavicle locking plate. The bone gap was filled with the cancellous and cortical bone graft harvested earlier. Finally, two 1.0 mm cerclage wires were inserted to hold the cortical graft to the clavicle shaft. Intra and post-operative healing was uneventful, and the patient was discharged on post-operative day two. A plain radiograph taken four months post-op revealed the presence of callus formation (Figure 6). He regained full shoulder range of motion with no functional limitation four months post-surgery and returned to work without shoulder pain after two momentous surgeries.

## Discussion

The distal radius is the most common graft source in upper limb surgical procedures. However, literature shows that olecranon graft can provide similar volumes of packed cancellous bone [4]. Generally, there are two available windows to harvest proximal ulna cortical and cancellous bone grafts: the dorsal cortical window (DSW) and the proximal cortical window (PSW) [5]. For DCW, a longitudinal skin incision around 2-3 cm was made over the dorsal proximal ulna to create an area of exposed cortex large enough to accommodate the osteotome. For the PCW technique, the same length skin incision was made more proximally over the posterior aspect of the olecranon, similar to the approach used to place the intramedullary rod. A split of 25 mm was made near the triceps tendon insertion to reach the proximal ulna. An area of exposed cortex, large enough to place our instrument, was prepared to create a cortical window. Here, the split triceps tendon needed repair. A cortical window was created with a 10 mm trephine, osteotome, or oscillating saw. The window was placed either 10 mm distal to the tip of the ulna (in case of DCW) or 5 mm volar to the tip (for PCW). After cortical window preparation, curettes were used to harvest the cancellous bone from the olecranon without violating the surrounding cortex. In our case, we approached the olecranon via the dorsal cortical window.

Olecranon bone grafts offer the following advantages: (1) Regional blocks, such as inter-scalene or axillary blocks, can be used for anaesthesia in ipsilateral upper limb surgery without necessitating general anaesthesia and endotracheal intubation; (2) for cancellous bone graft harvesting, cortical bone can be placed back into the bone window to prevent contour deformity and allow rapid healing; (3) periosteal repairs prevent haematoma formation due to reabsorption via interosseous circulation; (4) there are no vital structures such as nerves and arteries in the surgical field; (5) donor site scarring in olecranon bone grafts is minimal and located at a less conspicuous region; (6) post-operatively, the patient requires only simple arm sling immobilisation without plastic splints or casts [2].

Chim et al. documented a case series of 81 patients using olecranon bone grafts for scaphoid fracture fixation over eight years [3]. He reported no complications or pathological fractures of the olecranon following bone graft harvesting. Proper harvesting techniques can provide good quality but limited quantity of corticocancellous bone grafts. Jose and Kumar treated 20 patients with neglected forearm fractures using ipsilateral olecranon bone grafts for fracture fixation in a tertiary centre in India [6]. All patients were initiated on shoulder pendulum exercises and elbow and wrist range of motion exercises on day one post-operation. They regained the pre-injury functional status with complete fracture union around three months post-operatively. Micev et al. compared donor site morbidities between 44 patients with bone grafts harvested from the olecranon process or distal radius and revealed comparable patient (visual analogue scale questionnaire) and evaluator (elbow or wrist motion, presence of palpable defect, and radiographic analysis over defect at graft site)-determined outcomes with low risk of complications [7].

## Conclusions

Despite the lack of popularity, olecranon bone grafts remain one of the excellent options for obtaining cortical and cancellous bone in various surgical procedures as they can be easily obtained and cause no morbidity to patients. Olecranon bone grafts have emerged as a valuable resource in orthopaedic surgery due to their structural properties and clinical efficacy. Therefore, surgeons should prefer olecranon bone grafts as a source of autologous bone for routine surgical practice, given their proven robustness and reliability.
